# The effect of mindfulness-based cognitive therapy on rumination and a task-based measure of intrusive thoughts in patients with bipolar disorder

**DOI:** 10.1186/s40345-022-00269-1

**Published:** 2022-08-12

**Authors:** Jelle Lubbers, Dirk Geurts, Imke Hanssen, Marloes Huijbers, Jan Spijker, Anne Speckens, Mira Cladder-Micus

**Affiliations:** 1 Department of Psychiatry, Radboudumc Centre for Mindfulness, PO Box 9101, 6500 HB Nijmegen, The Netherlands; 2 Depression Expertise Centre, Pro Persona Mental Health Care, Nijmeegsebaan 61, 6525 DX Nijmegen, The Netherlands; 3 Behavioural Science Institute, Radboud University Nijmegen, PO Box 9104, 6500 HE Nijmegen, The Netherlands; 4 Donders Centre for Medical Neuroimaging, Donders Institute for Brain, Cognition and Behaviour, Radboud University Nijmegen, PO Box 9010, 6500 GL Nijmegen, The Netherlands

**Keywords:** Bipolar disorder, Mindfulness-based cognitive therapy, Breathing focus task, Depressive rumination, Positive rumination, Intrusive thoughts

## Abstract

**Background:**

Preliminary evidence suggests that Mindfulness-Based Cognitive Therapy (MBCT) is a promising treatment for bipolar disorder (BD). A proposed working mechanism of MBCT in attenuating depressive symptoms is reducing depressive rumination. The primary aim of this study was to investigate the effect of MBCT on self-reported trait depressive rumination and an experimental state measure of negative intrusive thoughts in BD patients. Exploratively, we investigated the effect of MBCT on positive rumination and positive intrusive thoughts.

**Methods:**

The study population consisted of a subsample of bipolar type I or II patients participating in a multicenter randomized controlled trial comparing MBCT + treatment as usual (TAU) (*N* = 25) to TAU alone (*N* = 24). Trait depressive rumination (RRS brooding subscale) and intrusive thoughts (breathing focus task (BFT)) were assessed at baseline (full subsample) and post-treatment (MBCT + TAU; *n* = 15, TAU; *n* = 15). During the BFT, participants were asked to report negative, positive and neutral intrusive thoughts while focusing on their breathing.

**Results:**

Compared to TAU alone, MBCT + TAU resulted in a significant pre- to post-treatment reduction of trait depressive rumination (*R*^2^ = .16, *F*(1, 27) = 5.15, *p* = 0.031; medium effect size (*f*^*2*^ = 0.19)) and negative intrusive thoughts on the BFT (*R*^2^ = .15, *F*(1, 28) = 4.88, *p* = 0.036; medium effect size (*f*^*2*^ = 0.17)). MBCT did not significantly change positive rumination or positive intrusive thoughts.

**Conclusions:**

MBCT might be a helpful additional intervention to reduce depressive rumination in BD which might reduce risk of depressive relapse or recurrence. Considering the preliminary nature of our findings, future research should replicate our findings and explore whether this reduction in rumination following MBCT indeed mediates a reduction in depressive symptoms and relapse or recurrence in BD.

**Supplementary Information:**

The online version contains supplementary material available at 10.1186/s40345-022-00269-1.

## Background

Bipolar disorder (BD) is an affective mental disorder characterized by a chronic course of recurrent depressive, (hypo)manic and/or mixed episodes. It is one of the leading causes of disability worldwide (Ferrari et al. 2016), causing high economic costs (Cloutier et al. 2018; Pini et al. 2005). BD patients suffer from mood symptoms half of their life, and depressive symptoms seem to predominate and contribute most to their disability (Judd et al. 2002). Despite the effectiveness of existing pharmacological and psychological interventions, in more than 40% of BD patients residual mood symptoms remain (Samalin et al. 2016). Therefore, it is of great importance to explore novel psychological interventions for BD to reduce these residual mood symptoms and prevent relapse at the longer term.

One of the key features of BD patients is their tendency to engage in maladaptive emotion regulation strategies such as depressive rumination, which they have in common with patients suffering from major depressive disorder (MDD) (Kovács et al. 2020; Silveira Jr and Kauer-Sant'Anna 2015; Ghaznavi and Deckersbach 2012). Depressive rumination can be described as the process of thinking perseveratively about one’s negative feelings and problems and their possible causes and consequences and is associated with depressive symptoms (Nolen-Hoeksema et al. 2008), also in BD (Johnson et al. 2008; Gucht et al. 2009). Moreover, there is considerable evidence that depressive rumination plays an important role in the onset of new depressive episodes and maintenance of MDD (Nolen-Hoeksema et al. 2008; Nolen-Hoeksema 2000; Michalak et al. 2011) and BD (Alloy et al. 2009; Gruber et al. 2011).

Mindfulness based cognitive therapy (MBCT) is an effective treatment for major depressive disorder (MDD) (Kuyken et al. 2016) that may sort its clinical effects by reducing depressive rumination (Velden et al. 2015). One of the proposed working mechanisms of MBCT is the increased ability to disengage from automatic maladaptive cognitive processes, such as depressive rumination. One of the core skills to be learned during MBCT is the ability to become aware of self-perpetuating ruminative thinking patterns and to let go of them. By becoming increasingly aware of automatic maladaptive cognitive processes and learning to decenter and disengage from them, patients prevent themselves to enter a vicious cycle of ruminative thinking that could otherwise aggravate symptoms of depression (Segal et al. 2013). As MDD and BD patients have many characteristics in common, such as the tendency to engage in ruminative thinking (Kovács et al. 2020; Ghaznavi and Deckersbach 2012), it has been hypothesized that MBCT may also be an effective treatment for BD (Deckersbach et al. 2012). Indeed, various systematic reviews concluded that MBCT seems promising as a treatment for BD, and that rumination may be an important therapeutic target (Xuan et al. 2020; Lovas and Schuman-Olivier 2018; Chu et al. 2018).

Two systematic reviews showed that MBCT reduced depressive rumination in MDD and that reduction of depressive rumination may mediate the reduction of depressive symptoms (Velden et al. 2015; Perestelo-Perez et al. 2017). The effect of MBCT on depressive rumination in BD is less well studied. An RCT that included 95 BD patients found a statistical trend towards reduced depressive rumination following MBCT (Perich et al. 2013). In addition, an open-label trial with 12 BD patients found depressive rumination to be significantly reduced after MBCT (Deckersbach et al. 2012).

In the context of an RCT on the effectiveness of MBCT in BD (Hanssen et al. 2019), we aimed to investigate the effect of MBCT on depressive rumination in BD patients. As traditional measures of rumination rely on self-report and therefore are prone to response and recall bias, the current study included both a self-report measure of trait rumination and an experimental measure, as suggested by van der Velden et al. (2015). We use the breathing focus task (BFT) (Hayes et al. 2010a; Hirsch et al. 2009) as an experimental state measure of negative, neutral, and positive intrusive thoughts patients report during a 5 min breathing exercise. The number of negative intrusive thoughts has previously been conceptualized as a state measure of depressive rumination (Southworth et al. 2017; Cladder-Micus et al. 2019). However, as negative intrusive thoughts measured by the BFT are not necessarily ruminative in nature, we will refer to the BFT as a state measure of negative, positive, and neutral intrusive thoughts for the remaining of this paper.

The main objective of the current study is to investigate the effect of MBCT in addition to treatment-as-usual (TAU) on depressive rumination and negative intrusive thoughts in BD. In addition, we aim to establish whether the experimental state measure of negative intrusive thoughts is related to a self-report measure of depressive symptoms and depressive rumination. Apart from depressive rumination, bipolar patients also engage in positive rumination, which has been defined as repetitively thinking about positive self-qualities and one’s current positive state (Feldman et al. 2008). However, little is known about the role of positive rumination in the course of BD (but, see (Gruber et al. 2011). Therefore, on an exploratory note, we assessed the relation between positive intrusive thoughts, manic symptoms, and positive rumination and assessed whether MBCT changed these variables.

## Methods

### Trial design

This study was part of a larger randomized, multicenter, evaluator-blinded, prospective clinical trial assessing the clinical effectiveness of MBCT as an additional treatment for BD (Hanssen et al. 2019). In total, 144 patients were randomly assigned to an 8-week MBCT training in combination with treatment as usual (TAU) or TAU only. Assessments were conducted up to 15 months follow-up. For the current study, baseline and post-treatment assessments were used.

### Participants and procedure

The following inclusion criteria were applied: (a) age ≥ 18; (b) SCID-I confirmed diagnosis of bipolar type I or II; (c) suffered from at least (i) two lifetime depressive episodes (current or in (partial) remission), and (ii) one affective episode within the year prior to baseline; (d) No current (hypo)manic episode (young mania rating scale score < 12) (Young et al. 1978). Exclusion criteria were: (a) manic episode within the last 3 months before start of the trial; (b) lifetime diagnosis of schizophrenia or schizoaffective disorder, current substance abuse disorder, organic brain syndrome, antisocial or borderline personality disorder; (c) risk of suicide or aggression; (d) presence of a concurrent medical conditions impeding the ability to participate.

Participants were recruited from seven specialized outpatient clinics for adults with BD. Participants received a letter from their attending clinicians that informed them about the study. After verbal consent was obtained, participants were screened by phone to assess eligibility and detailed information about the study was provided. When interested, possible eligible patients were invited for a research interview (T0) that was conducted by a trained research assistant. In order to keep the duration of the interview at acceptable limits, patients were asked to complete a set of (online) self-report questionnaires (T0a) at home, which included the brooding subscale of the Ruminative Response Scale (RRS; (Treynor et al. 2003)) and the Responses to Positive Affect–Dutch Version (RPA-NL; (Feldman et al. 2008)). During the interview patients were extensively checked for in- and exclusion criteria using the structural clinical interview for DSM-IV (SCID; (First et al. 1996)) and checked in accordance with DSM-V criteria (Diagnostic et al. 2013), and written informed consent was obtained. In addition, the interview at T0 also included the Inventory of Depressive Symptomatology–Clinician administered (IDS-C; (Akkerhuis and Vertaling 1997)), and the Young Mania Rating Scale (Young et al. 1978). At the end of the interview, patients that did not yet completed the self-report questionnaires (T0a) were requested to do this as soon as possible, resulting in (*Mdn* (P25; P75) = − 1 (− 6; 6) days between T0a and T0. After the interview, due to practical reasons (laptop not available, lack of research assistance, time schedule too tight), only a subset of participants (74 out of 144; see Additional file 1: Figure S1) were invited to perform a set of cognitive tasks at baseline (T0b) and at post-treatment (T1b), including the breathing focus task (BFT; see paragraph ‘Measures’). Twenty-two of those invited did not participate because they refused (*n* = 8), could not be planned anymore (*n* = 9) or because of other practical reasons (*n* = 5). Sociodemographic characteristics of the 52 participants who performed the experimental tasks were neither significantly different from the 22 participants who were invited but not participated (Additional file 2: Table S1), nor from the total group of 92 participants who did not perform the tasks (Additional file 2: Table S2). Research assistants who conducted the assessments were blind for treatment allocation. At baseline, the cognitive tasks (T0b) were administered (*Mdn* (P25; P75) = 13 (8; 28.5) days after the interview (T0) and (*Mdn* (P25; P75) = 9 (15; 29) days after the self-report questionnaires (T0a). At posttreatment, these time differences were as follows (*Mdn* (P25; P75): self-report questionnaires (T1a) 4 (− 13; 17) days after interview (T1), cognitive tasks (T1b) 19.5 (0; 37) days after interview (T1) and 17.5 (− 1; 32) days after self-report questionnaires (T1a). In addition, the post-treatment interview (T1) took place 18.7 (15.4; 21.4) weeks after the baseline interview (T0), the self-report questionnaires (T1a) were administered 19.6 (16.1; 21.6) weeks after T0a, and the cognitive tasks (T1b) were administered 16.9 (15.0; 21.3) weeks after T0b.

## Measures

### Inventory of depressive symptomatology—clinician administered (IDS-C)

The IDS-C is an observer-rated 30-item questionnaire that assesses the severity of depressive symptoms (range 0–84) over the past week (Akkerhuis and Vertaling 1997). The IDS-C has good psychometric qualities (Rush et al. 1996; Trivedi et al. 2004) and was administered by trained research assistants (Hanssen et al. 2019).

### Young mania rating scale (YMRS)

The YMRS is a reliable, valid and sensitive 11-item questionnaire that assesses the severity of (hypo)manic symptoms (range 0–60) (Young et al. 1978) and was administered by trained research assistants.

### Brooding subscale of ruminative response scale—extended version (RRS-EXT)

The brooding subscale of the RRS consists of 5 items that assesses a self-report measure of brooding (range 5–20): a form of rumination strongly related to levels of depression (Treynor et al. 2003). The previously reported adequate internal consistency (*α* = 0.77) is comparable to consistency in the current sample (*α* = 0.81).

### Self- and emotion-focused subscales of responses to positive affect—dutch version (RPA-NL)

The self-focused (4 items; range 4–16) and emotion-focused (5 items; range 5–20) subscales of the RPA assess levels of self-focused and emotion-focused positive rumination. The RPA-NL (Feldman et al. 2008) has shown satisfactory internal consistency (Raes et al. 2009a) which was also found in the current sample (self-focused: *α* = 0.86; emotion focused: *α* = 0.77).

### Breathing focus task (BFT)

The BFT, originally developed by Borkovec and colleagues (Borkovec et al. 1983), is considered to be an experimental measure of intrusive thoughts (Hayes et al. 2010a; Hirsch et al. 2009; Cladder-Micus et al. 2019). Generally, the BFT consists of a first assessment phase followed by a worry or negative mood induction phase and a second assessment phase. Due to ethical concerns regarding inducing negative mood in a clinical sample, the BFT has also been conducted with the first assessment phase only (Cladder-Micus et al. 2019). As our BD sample also included clinically depressed patients, this last version of Cladder-Micus et al. (2019) was used.

The BFT consisted of a practice phase and the actual task. During the practice phase, participants were asked to practice focusing on their breathing for 20 s. After that, participants were asked to concentrate on their breathing for 45 s, while noticing distracting intrusive thoughts. During this period, a computer-generated tone sounded 3 times at random intervals of 10–20 s. After each tone, participants verbally reported whether they were focused on their breathing or distracted by an intrusive thought. When distracted by a thought, participants reported a short word label (e.g. “cannot concentrate”) and classified the thought as negative, positive or neutral. When participants were focused on their breathing, they responded by saying ‘breath’ (Dutch: ‘adem’). During the actual task, participants were asked to focus on their breathing for 5 min and responded to 12 tones at random intervals of 20–30 s analogously to the practice phase.

After the 5 min breathing period, participants were asked to fill in a self-report measure of (i) ‘percentage of time distracted by ruminating (Dutch = “piekeren”) thoughts’ (VAS, 0–100%), (ii) ‘percentage of time distracted by positive thoughts’ (VAS, 0–100%), (iii) ‘percentage of time focused on breathing’ (VAS, 1–100%), (iv) ‘how difficult it was to focus on breathing’ (very difficult–not at all difficult).

## Intervention

### Mindfulness-based cognitive therapy (MBCT)

Patients were randomly assigned to either (i) MBCT + TAU, in which patients received MBCT in addition to usual care typically consisting of pharmacotherapy, psycho-education and self-management interventions, or (ii) TAU alone. MBCT offered in the current study is based on the manual developed for relapse prevention in unipolar depression (Mindfulness-based cognitive therapy for depression 2002), and was slightly adapted to address the needs of BD patients (Hanssen et al. 2019). The MBCT training consisted of 8 weekly sessions of 2.5 h, one 6 h silent day, and daily home practice (± 45 min). MBCT was taught by a therapist with knowledge of BD together with a MBCT teacher meeting the advanced criteria of the Association of Mindfulness Based Teachers in the Netherlands and Flanders (Belgium) which are in concordance with the Good Practice guidelines of the UK Network of Mindfulness-Based Teacher Trainers (Crane et al. 2012).

## Statistical analyses

### Depressive symptoms, depressive rumination and negative intrusive thoughts

Data were analyzed by using the SPSS 25.0 software package and visualized by R, and Graphpad Prism version 8.0. The main goal of the current study was to get more mechanistic insight into the relation between depressive symptoms, depressive rumination and negative intrusive thoughts, and whether MBCT could change those variables in BD. Therefore, we performed per protocol analyses for the pre-post data: participants from the MBCT + TAU group who received a minimum effective dose of 4 or more sessions were included, as proposed by Teasdale et al. (2000) (Teasdale et al. 2000). At first, demographic variables and baseline scores were compared between the conditions using independent sample t-tests, Mann–Whitney tests, Fisher’s Exact test, and χ^2^ tests respectively. Secondly, spearman’s rho correlations were used to evaluate the association of negative intrusive thoughts on the BFT at baseline with depressive symptoms, depressive rumination, and the self-reported time patients were distracted by ruminating thoughts. In addition, to check whether the time difference in administration of the IDS-C (interview; T0), RRS brooding subscale (T0a) and BFT (T0b) influenced the associations between those measures, analysis of variance was performed with time between T0/T0a/T0b (and interactions) as predictors, e.g. depressive symptoms was entered as dependent variable while (i) negative intrusive thoughts and (ii) time between T0 (IDS-C) and T0b (BFT), and an interaction term, were entered as predictors. Thirdly, the effect of MBCT on depressive symptoms was assessed in the current sample by repeated measures analysis of variance (rmANOVA), with time (baseline to post-treatment) as within subject factor and group (MBCT + TAU vs TAU) as between subject factor. Fourth, the effect of MBCT on depressive rumination and intrusive thoughts was assessed by bootstrap linear regression analysis. This type of analysis was chosen because count data from the BFT was positively skewed, and although the (negative) binomial distribution is commonly used for positively skewed count data, these distributions did not adequately fit our data. Change in (i) RRS brooding score, and (ii) number of negative, positive, neutral and total intrusive thoughts (post-treatment—baseline) was entered as dependent variable while group (MBCT + TAU vs TAU) was entered as predictor. Bias corrected and accelerated (BCa) 95% confidence intervals (CI’s) and significance values were calculated based on 5000 bootstrap samples. Because bootstrap analysis does not rely on assumptions of normality or homoscedasticity, they provide us with an accurate estimate of the unstandardized regression coefficient *B* for group as a predictor variable. Cohens *f*^2^, the standard effect size measure for linear regression, was calculated by the following equation:$f^{2} = \frac{{R^{2} }}{{1 - R^{2} }}$, in which f^2^ ≥ 0.02, f^2^ ≥ 0.15 and f^2^ ≥ 0.35 represent small, medium, and large effect sizes (Cohen 1977). The bootstrapped linear regression models were run 5 times to confirm robustness of the output.

### Manic symptoms, positive rumination, and positive intrusive thoughts

On an exploratory note we first explored with spearman’s rho correlations whether there is an association between baseline positive intrusive thoughts, manic symptoms, positive rumination, and self-reported time distracted by positive thoughts. In addition, to check whether the time difference between interview (T0), self-report questionnaires (T0a) and BFT (T0b) influenced those results, analysis of variances were run in a similar way as described for depressive symptoms, RRS brooding score, and negative intrusive thoughts. Secondly, we assessed the effect of MBCT on manic symptoms using rmANOVA. Third, we explored the effect of MBCT on emotion-focused and self-focused positive rumination by bootstrap linear regressions, with change in those variables as dependent variable, while group was entered as predictor variable.

## Results

### Sample characteristics

From the 52 participants who performed the BFT at baseline, three were excluded from analyses due to missing data (*n* = 2) or because the valence was not reported for > 33% of the generated tones (*n* = 1). Thus, BFT data was available for 49 participants at baseline (MBCT + TAU, *N* = 25; TAU, *N* = 24). Of these participants, 34 (69%) completed the BFT post-treatment (not significantly different in terms of sociodemographics and clinical characteristics from non-completers; Additional file 2: Table S3), of which 32 participated in at least 4 MBCT sessions. Two participants were excluded from pre-post analyses because the valence was not reported for > 33% of the generated tones post-treatment, resulting in a final sample of 30 participants equally divided over the MBCT + TAU (*N* = 15) and TAU group (*N* = 15).

Sociodemographic and clinical characteristics, and baseline scores on outcome measures are presented in Table 1. At baseline, participants had mild depressive symptoms and were in remission of manic symptoms. Participants in the MBCT + TAU group showed higher levels of self-focused and emotion-focused positive rumination than the TAU group. There were no other significant differences between both groups.

**Table 1:** Sociodemographic and clinical characteristics, and baseline outcome measures and the breathing focus task at baseline

	Total at baseline (*N* = 49)	MBCT + TAU (*N* = 15)	TAU (*N* = 15)	MBCT + TAU vs TAU	
				Test statistic	*p*
Demographic characteristics					
Age, *Med* (P25-P75)	48.5 (37.5–54.0)^1^	52.0 (43.5–54.0)	45.0 (31.0–51.5)	*U* = 81.5, *z* =− 1.29	0.20^c^
Gender, *Female* (%)	66.7^1^	60	66.7	χ^2^(1) = 0.14	1.00^a^
Education					0.84^b^
Low (%)	12.5	6.7	6.7		
Medium (%)	29.2	26.7	40		
High (%)	58.3	66.7	53.3		
Married/living together (%)	54.2^1^	46.7	73.3	χ^2^(1) = 2.22	0.14^b^
Employed	41.7^1^	53.3	26.7	χ^2^(1) = 2.22	0.14^a^
Clinical characteristics					
Bipolar type I (%)	61.2	60	66.7	χ^2^(1) = 0.14	1.00^a^
Age first episode, *Med* (P25-P75)	20.0 (17.0–23.0)	23.0 (19.5–27.0)	18.0 (17.5–22.0)	*U* = 68.0, *z* =− 1.86	0.067^c^
Number of episodes, *Med* (P25-P75)	13.0 (7.0–40.0)	12.0 (6.5–47.50)	10.0 (8.5–18.5)	*U* = 108, *z* =− 0.19	0.87^c^
Outcome measures					
Depressive symptoms (IDS-C), *Med* (P25-P75)	12.0 (5.0–24.0)	13.0 (6.5–28.5)	12.0 (5.0–24.0)	*U* = 102, *z* =− 0.44	0.68^c^
Manic symptoms (YMRS), *Med* (P25-P75)	1.0 (0.0 – 3.0)	1.0 (0.0–4.0)	1.0 (0.0–2.0)	*U* = 101, *z* =− 0.52	0.62^c^
Depressive rumination (RRS_br), *Mean (SD)*	11.5 (3.5)^4^	11.8 (3.8)^1^	11.1 (3.6)	*t*(27) =− 0.52	0.61^d^
Emotion-focused positive rumination (RPA_ER), *Mean (SD)*	13.1 (2.8)^4^	14.7 (2.3)^1^	12.5 (2.3)	*t*(27) =− 2.59	0.015^d^
Self-focused positive rumination (RPA_SR), *Mean (SD)*	8.8 (2.8)^4^	10.5 (2.1)^1^	8.2 (2.5)	*t*(27) =− 2.66	0.013^d^
Breathing focus task	*Med* (P25-P75)	*Med* (P25-P75)	*Med* (P25-P75)		
Total intrusive thoughts	5.0 (3.0–7.0)	3.0 (2.0–7.0)	5.0 (3.0–7.0)	*U* = 104, *z* =− 0.38	0.71^c^
Negative intrusive thoughts	1.0 (0.0–2.0)	0.0 (0.0–2.5)	1.0 (0.0–1.0)	*U* = 107, *z* =− 0.27	0.81^c^
Positive intrusive thoughts	1.0 (0.0–2.0)	1.0 (0.0–2.0)	1.0 (0.0–2.0)	U = 111, *z* =− 0.064	0.97^c^
Neutral intrusive thoughts	2.0 (0.0–4.0)	1.0 (0.0 2.5)	2.0 (1.0–4.5)	*U* = 81.5, *z* =− 1.32	0.20^c^
Percentage of time distracted by ruminating thoughts (VAS)	22.5 (6.0–42.0)^2^	16.0 (1.0–34.0)^2^	22.0 (13.5–36.5)	*U* = 79.5, *z* =− 0.83	0.41^c^
Percentage of time distracted by positive thoughts (VAS)	36.0 (19.0–52.0)^2^	19.0 (2.0–45.0)^2^	36.0 (24.0–53.5)	*U* = 56.0, *z* =− 1.92	0.058^c^

*IDS-C *inventory of depressive symptomatology—clinician administered, *YMRS *young mania rating scale

*RRS_br *brooding subscale of ruminative response scale, *RPA_ER *emotion-focused subscale of the responses to positive affect questionnaire, *RPA_SR *self-focused subscale of the responses to positive affect questionnaire

*VAS *visual analogue scale

^a^χ^2^ test

^b^Fisher’s Exact test

^c^Mann–Whitney test

^d^Independent samples t test

^1,2,3^Number of missing values: ^1^1 missing, ^2^2 missing, ^3^3 missing

### Correlations between depressive symptoms, trait rumination, and the breathing focus task at baseline

The number of negative intrusive thoughts was significantly correlated with the self-reported time participants were distracted by ruminating thoughts (*rs*(44) = 0.53, *p* = 0.001), and trait rumination was significantly correlated with depressive symptoms (*rs*(43) = 0.57, *p* < 0.0001). Of note, no significant correlation was found between the number of negative intrusive thoughts with trait rumination (*rs*(43) = 0.25, *p* = 0.10) nor with depressive symptoms (*rs*(47) = 0.17, *p* = 0.25). In addition, analysis of variance showed that the time between administration of those measures did not influence the associations found between those measures (Additional file 2: Table S4).

### Effect of MBCT on depressive symptoms

Before analyzing the effect of MBCT on rumination and negative intrusive thoughts on the BFT, we first assessed the effect of MBCT on depressive symptoms in this subsample. No significant beneficial effect of MBCT + TAU compared to TAU was found on depressive symptoms (Time × Group: *F*(1, 28) = 0.72, *p* = 0.40, η^2^ = 0.025), which is in line with the results of the overarching RCT (Hanssen et al. 2021, submitted).

### Effect of MBCT on trait rumination

**Fig. 1:**
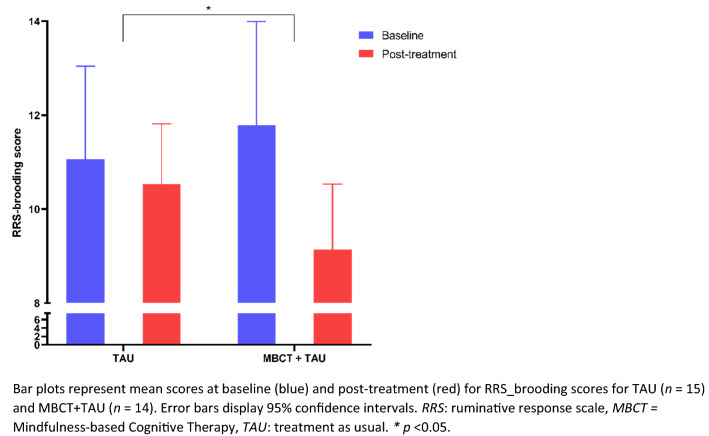
Effect of mindfulness-based cognitive therapy on trait depressive rumination over time

Then, we investigated whether group (MBCT + TAU compared to TAU) predicted a change in the questionnaire-based measure of depressive rumination and the number of negative intrusive thoughts on the BFT. Table 2 shows bootstrapped BCa 95% confidence intervals for all conducted linear regression models. Receiving MBCT + TAU compared to TAU resulted in a decrease of 2.05 points on the RRS brooding subscale from baseline to post-treatment (*R*^2^ = 0.16, *F*(1, 27) = 5.15, *p* = 0.031) with a medium effect size (*f*^*2*^ = 0.19), see Fig. 1.

**Table 2:** Bootstrapped Bias-corrected and Accelerated 95% confidence intervals of B for regression equations regarding change scores of depressive rumination, intrusive thoughts on the breathing focus task and positive rumination

	*B*	Bias	SE	p-value	BCa 95% CI of *B*	
					Lower	Upper
Broodings subscale of RRS: RRS_br.T1–RRS_br.T0 = group + intercept						
Constant	1.58	− 0.005	1.32	0.25	− 1.09	4.19
Group (MBCT + TAU vs TAU)	− 2.11	0.005	0.92	0.035	− 3.93	− 0.33
Negative intrusive thoughts on the BFT: BFT.neg.T1–BFT.neg.T0 = group + intercept						
Constant	2.00	0.005	1.00	0.064	0.20	3.98
Group (MBCT + TAU vs TAU)	− 1.47	− 0.003	0.64	0.033	− 2.76	− 0.26
Neutral intrusive thoughts on the BFT: BFT.neutral.T1–BFT.neutral.T0 = group + intercept						
Constant	− 2.60	− 0.035	1.17	0.055	− 4.93	− 0.47
Group (MBCT + TAU vs TAU)	1.93	0.023	0.84	0.047	0.29	3.78
Positive intrusive thoughts on the BFT: BFT.pos.T1–BFT.pos.T0 = group + intercept						
Constant	− 0.13	− 0.005	1.32	0.92	− 2.73	2.38
Group (MBCT + TAU vs TAU)	0.33	0.007	1.01	0.75	− 1.61	2.55
Total intrusive thoughts on the BFT: BFT.total.T1–BFT.total.T0 = group + intercept						
Constant	− 0.73	0.023	1.60	0.67	− 3.94	2.52
Group (MBCT + TAU vs TAU)	0.80	− 0.009	1.17	0.51	− 1.48	3.07
Emotion-focused subscale of RPA: RPA_ER.T1–RPA_ER.T0 = group + intercept						
Constant	0.71	− 0.013	1.44	0.63	− 1.80	3.31
Group (MBCT + TAU vs TAU)	− 0.18	0.009	0.80	0.82	− 1.93	1.56
Self-focused subscale of RPA: RPA_SR.T1–RPA_SR.T0 = group + intercept						
Constant	2.39	0.015	1.45	0.11	− 0.55	5.36
Group (MBCT + TAU vs TAU)	− 1.59	− 0.006	0.94	0.11	− 3.40	0.23

*B *unstandardized regression coefficient, *SE *standard error, *BCa *bias-corrected and accelerated

*BFT *breathing focus task, *RRS *ruminative response scale, *RPA *responses to positive affect, *T0 *baseline

*T1 *post-treatment, *MBCT *mindfulness-based cognitive therapy, *TAU *treatment as usual

### Effect of MBCT on intrusive thoughts on the BFT

**Fig. 2:**
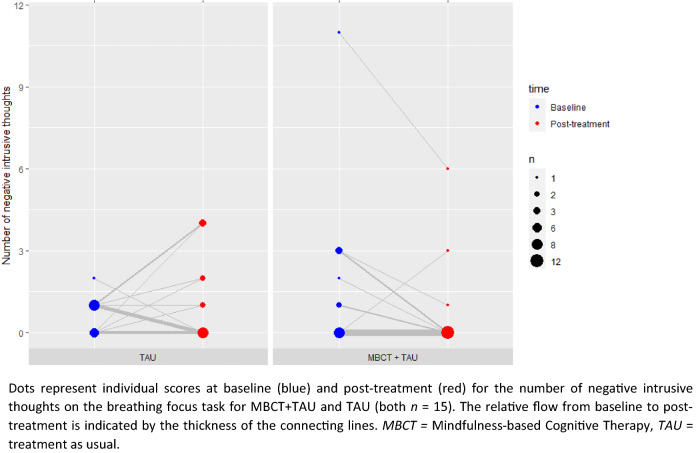
Effect of mindfulness-based cognitive therapy on negative intrusive thoughts over time

Next, we conducted linear regression models for the BFT for each valence separately, and for the total number of intrusive thoughts. MBCT + TAU compared to TAU alone resulted in a decrease in the state measure of negative intrusive thoughts: (*R*^2^ = 0.15, *F*(1, 28) = 4.88, *p* = 0.036) with a medium effect size (*f*^*2*^ = 0.17). Individual data points show that the majority of participants receiving MBCT + TAU show a decrease in negative intrusive thoughts from baseline to post-treatment, while this is not the case for participants receiving TAU only (Fig. 2).

Receiving MBCT + TAU compared to TAU alone resulted in an increase in neutral intrusive thoughts (*R*^2^ = 0.15, *F*(1, 28)  = 5.09, *p* = 0.032) with a medium effect size (*f*^*2*^ = 0.18). No effect of MBCT on positive (*R*^2^ = 0.004, *F*(1, 28) = 0.11, *p* = 0.74, *f*^*2*^ = 0.004), or total intrusive thoughts (*R*^2^ = 0.016, *F*(1, 28) = 0.45, *p* = 0.51, *f*^*2*^ = 0.016) was found.

### Exploratory analyses: manic symptoms, positive rumination and positive intrusive thoughts

The number of positive intrusive thoughts was associated with the self-reported time participants were distracted by positive thoughts (*rs*(44) = 0.46, *p* = 0.001), but not with manic symptoms nor with emotion-focused (*rs*(44) = 0.12, *p* = 0.42) or self-focused (*rs*(44) = 0.03, *p* = 0.87) positive rumination. In addition, in this subsample no beneficial effects were found on manic symptoms (Time × Group: *F*(1, 28) = 0.023, *p* = 0.88, η^2^ = 0.001), which is in line with the overall RCT (Hanssen et al. 2021, submitted). Moreover, no effect of MBCT + TAU compared to TAU was found on questionnaire-based measures of positive rumination (RPA_ER: *R*^2^ = 0.002, *F*(1, 27) = 0.45, *p* = 0.83, *f*^*2*^ = 0.002; RPA_SR: *R*^2^ = 0.093, *F*(1, 27) = 2.78, *p* = 0.11, *f*^*2*^ = 0.09).

## Sensitivity analysis

When the linear regression models were run another four times, bootstrapped BCa 95% confidence intervals and corresponding p-values for *B* were comparable, indicating robustness of these results (Additional file 2: Tables S5–11).

## Discussion

In the context of an overarching RCT investigating the efficacy of MBCT for BD, we found that MBCT significantly reduced self-reported trait depressive rumination and the experimental measure of negative intrusive thoughts in BD patients. To our best knowledge, this is the first study to use both an experimental (state) measure of intrusive thoughts and a self-report (trait) measure of depressive rumination in BD. Both trait rumination (the general tendency to ruminate) and the number of negative intrusive thoughts on the BFT were affected by MBCT, supporting the assumption that MBCT changes dysfunctional cognitive patterns such as depressive rumination, also in BD.

Our current finding that MBCT reduced depressive rumination in BD patients is in line with previous controlled studies in MDD showing reductions in trait depressive rumination (Velden et al. 2015; Perestelo-Perez et al. 2017; Cladder-Micus et al. 2019). The effect of MBCT on trait rumination in BD is less well studied, yet, available evidence points in the same direction (Deckersbach et al. 2012; Perich et al. 2013). A statistical trend towards reduced depressive rumination after MBCT was observed in an RCT that included 95 BD patients (Perich et al. 2013), and depressive rumination was significantly reduced following MBCT in an open label trial with 12 BD patients (Deckersbach et al. 2012).

To put the reduction in rumination through MBCT in perspective, we nominally compare our data to the study of Raes et al. (2009b), where never depressed controls on average scored 9.3 (*SD* = 2.9) and remitted patients on average 11.0 (*SD* = 3.0) on the RRS brooding scale. Comparing these scores to those reported in the current study (from pre- 11.8 (SD = 3.8) to post-MBCT 9.1 (SD = 2.4)) suggests that MBCT decreased rumination in our sample to levels comparable to never depressed controls (Raes et al. 2009b).

We also found a reduction of negative intrusive thoughts on the BFT, which is in line with a controlled study in MDD showing a reduction of negative intrusive thoughts after MBCT (Cladder-Micus et al. 2019). To our knowledge, the effect of MBCT on negative intrusive thoughts, or other state measures of negative thinking have not been studied in BD to date. Such an experimental state measure provides added value to self-report trait measures as it provides information regarding MBCT-induced changes in ‘on-line’ experience of negative intrusive thoughts during task performance, and is therefore less susceptible for recall and response bias (Veenman 2011). Thus, our findings indicate that MBCT reduced both the general tendency to ruminate and also reduced negative intrusive thoughts during an experimental task.

Of interest, MBCT reduced trait rumination and negative intrusive thoughts in a relatively euthymic sample, even without a significant effect on depressive symptoms. This reduction in depressive rumination and negative intrusive thoughts may be beneficial for the course of bipolar disorder, as has been reported in an uncontrolled study of MBCT in MDD showing that post-treatment levels of depressive rumination predicted the risk of relapse in a 12-month follow-up period, even when controlled for previous numbers of depressive episodes and residual depressive symptoms (Michalak et al. 2011). In addition, also in bipolar disorder depressive rumination seems to be involved in the onset of new depressive episodes (Alloy et al. 2009) and was associated with greater lifetime depression frequency (Gruber et al. 2011). Thus, our data provides preliminary evidence showing that MBCT reduces depressive rumination in BD even in relatively euthymic patients, which potentially reduces the risk on depressive relapse or recurrence.

We were also interested in the extent to which the state measure of negative intrusive thoughts relates to the trait measure of depressive rumination. Interestingly, we did not find the number of negative intrusive thoughts on the BFT to be significantly correlated with trait depressive rumination. This null-finding may be explained by our small sample size. In addition, the time between administration of the BFT (T0b) and the RRS brooding subscale (T0a) could have lowered the likelihood of finding an association. However, analysis of variance did not reveal a significant interaction between the RRS brooding subscale and the time between those measures (T0a and T0b) on explained variance in negative intrusive thoughts. Moreover, it could also be that negative thoughts on the BFT are in fact not all ruminative and intrusive in nature and therefore do not always correspond to ruminative depressogenic thoughts. Previous work on the BFT in chronically depressed patients indeed showed similar results: no relationship was found between the RRS_brooding subscale and the number of negative intrusive thoughts (Cladder-Micus et al. 2019). In addition, state measures, such as negative intrusive thoughts on the BFT are much more affected by situational cues. Thus, our state measure of negative intrusive thoughts and our trait measure of depressive rumination may be different constructs that may both change over time, but this change is not necessarily related and does not per se happen simultaneously.

As secondary objective we exploratively investigated the effect of MBCT on positive rumination. MBCT did not change self-reported trait positive rumination nor positive intrusive thoughts on the BFT. These findings may be caused by the fact that most patients in our sample were in remission of manic symptoms (showing very low scores on the YMRS), resulting in floor effects. In addition, positive intrusive thoughts on the BFT may not entirely reflect (hypo)manic intrusive thoughts. BD patients might not appraise (hypo)manic thoughts as positive and may therefore not report them as positive on the BFT. Thus, this null-finding warrants further investigation.

### Strengths, limitations and future research

One major strength is the innovative character of this study. This is the first study to our knowledge that includes, apart from conventional self-report measures, an experimental measure to assess the effect of MBCT on rumination and intrusive thoughts in BD. The use of an additional experimental measure provides complementary knowledge triangulating (Noble and Heale 2019) information derived by questionnaires (Velden et al. 2015). Here, we showed that both measures were affected by MBCT, strengthening the assumption that MBCT reduces depressive rumination and negative thinking in BD.

The most important limitation of this study is the relatively small sample size. Therefore, the current results should be replicated in larger samples. In addition, measures of depressive and manic symptoms (T0 interview), self-report questionnaires (T0a) and the BFT (T0b) were not administered at the same day, which may have weakened the associations at baseline. However, when including the difference in time between measures, we did not find a significant influence (interaction) of time on explained variance for all those measures. Another limitation is the absence of an active control group, which prevents drawing conclusions on what specific aspects of the MBCT may have contributed to the reduction of depressive rumination and negative intrusive thoughts. Another point of attention is the BFT itself, that may yet need refinement. In this study all reported BFT scores fall within the lower regions (0–3) of the measurement tool, with many participants reporting 0 negative intrusive thoughts. The zero-inflated outcomes observed on the BFT in this and other studies (Hayes et al. 2010a; Hirsch et al. 2009; Cladder-Micus et al. 2019; Hoorelbeke et al. 2015; Hayes et al. 2010b; Ikani et al. 2021) might prevent finding a relation with trait measures and may restrict the interpretation of the BFT outcomes. Future studies might benefit from reintroducing the worry/rumination induction which has been previously used in context of the BFT (Hayes et al. 2010a; Hirsch et al. 2009; Hayes et al. 2010b) or extending the duration of the task (e.g. 24 beeps in 10 min). However, despite the relatively low scores on the BFT, in our study the BFT was sensitive to pick up MBCT-induced reductions of negative intrusive thoughts. Lastly, one of the key components of MBCT is that participants are specifically trained to pay attention to and become aware of their breathing (mindful breathing). One could speculate that patients receiving MBCT are therefore less easily distracted by and would report less intrusive thoughts in general, irrespective of valence. However, in line with previous research (Cladder-Micus et al. 2019) we only found a reduction in negative intrusive thoughts after MBCT. In addition, an increase in neutral intrusive thoughts and no effect on total intrusive thoughts was observed. This might be explained by MBCT leading to a reinterpretation of thoughts with a negative content as neutral thoughts. For future research it seems valuable to include a self-report measure of state rumination to relate to the BFT, as for example the Brief State Rumination Inventory (BSRI) (Marchetti et al. 2018).

The current study is based on a pre-post design, which prevents conclusions on whether change in depressive rumination or negative intrusive thoughts precedes a change in depressive symptoms or is related to depressive relapse. To investigate whether depressive rumination is a mediator of the effect of MBCT on depressive symptoms and depressive relapse in BD, future well-powered longitudinal studies including multiple time points are required (Kazdin 2007). A better understanding of the underlying mechanisms in the beneficial effects of MBCT may eventually provide insight into the individual differences in effectiveness, and may help to improve effectiveness of MBCT.

## Conclusion

MBCT might be of added value to usual treatment of BD to reduce depressive rumination, a known risk-factor for relapse in BD (Alloy et al. 2009; Gruber et al. 2011). Moreover, our data suggest that current state and trait measures of negative thinking and depressive rumination respectively carry mutually independent information. Future studies should assess whether the additive information that state measures carry are indeed of predictive value for clinical improvement in terms of symptom reduction as well as depressive relapse.

## Supplementary Information

**Additional file 1:** **Figure S1. CONSORT Flow Diagram** *Practical reasons consisted of limited number of research laptops; no research assistant available at every outpatients clinic, not enough time between baseline measurement and start MBCT.

**Additional file 2:** **Table S1.** Sociodemographic and clinical characteristics, and baseline measures for patients that agreed or refused to perform the experimental tasks. **Table S2.** Sociodemographic and clinical characteristics, and baseline measures for patients who performed, or did not perform the experimental tasks. **Table S3.** Sociodemographic and clinical characteristics, and baseline measures for patients who completed baseline and post-treatment BFT versus patients who completed baseline BFT only. **Table S4.** No influence of time between measures of depressive/manic symptoms (T0), self-report questionnaires (T0a), and the BFT scores (T0b), on the association between those measures at baseline. **Table S5.** Bootstrapped BCa 95% confidence intervals of B for the regression model regarding the brooding subscale of the RRS: RRS_br.T1 – RRS_br.T0 = group + intercept. **Table S6.** Bootstrapped BCa 95% confidence intervals of B for the regression model regarding the number of negative intrusive thoughts: BFT.neg.T1 – BFT.neg.T0 = group + intercept. **Table S7.** Bootstrapped BCa 95% confidence intervals of B for the regression model regarding the number of positive intrusive thoughts: BFT.pos.T1 – BFT.pos.T0 = group + intercept. **Table S8.** Bootstrapped BCa 95% confidence intervals of B for the regression model regarding the number of neutral intrusive thoughts: BFT.neutral.T1 – BFT.neutral.T0 = group + intercept. **Table S9.** Bootstrapped BCa 95% confidence intervals of B for the regression model regarding the number of total intrusive thoughts: BFT.total.T1 – BFT.total.T0 = group + intercept. **Table S10.** Bootstrapped BCa 95% confidence intervals of B for the regression model regarding the emotion-focused subscale of the RPA: RPA_ER.T1 – RPA_ER.T0 = group + intercept. **Table S11.** Bootstrapped BCa 95% confidence intervals of B for the regression model regarding the self-focused subscale of the RPA: RPA_SR.T1 – RPA_SR.T0 = group + intercept

## Data Availability

The dataset used and/or analyzed for the current study are available from the corresponding author on reasonable request.

## Abbreviations

BD: Bipolar disorder
BFT: Breathing focus task
IDS-C: Inventory of depressive symptomatology—clinician administered
MDD: Major depressive disorder
MBCT: Mindfulness-based cognitive therapy
rmANOVA: Repeated measures analysis of variance
RRS: Ruminative response scale
RRS_br: Brooding subscale of the ruminative response scale
RPA: Responses to positive affect
RPA_EM: Emotion-focused subscale of the responses to positive affect
RPA_SF: Self-focused subscale of the responses to positive affect
TAU: Treatment as usual
VAS: Visual analogue scale
YMRS: Young mania rating scale
