# Supplementary figures and images for: The effect of mindfulness-based cognitive therapy on rumination and a task-based measure of intrusive thoughts in patients with bipolar disorder
Source: Int J Bipolar Disord. 2022 Aug 12;10:22. doi: 10.1186/s40345-022-00269-1 (PMC9374865; doi:10.1186/s40345-022-00269-1)

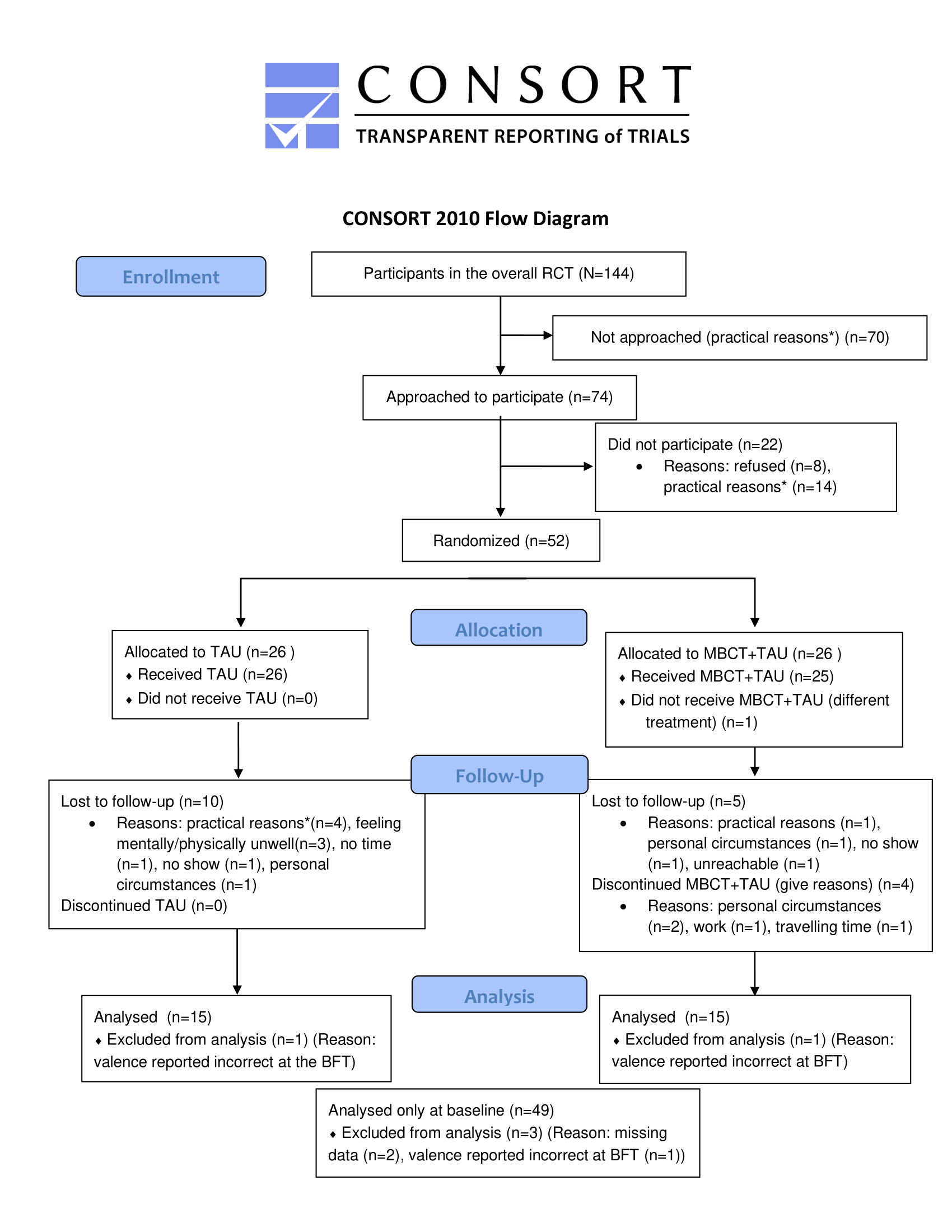

Supplement: Supplementary file 1 — Additional file 1: Figure S1. CONSORT Flow Diagram *Practical reasons consisted of limited number of research laptops; no research assistant available at every outpatients clinic, not enough time between baseline measurement and start MBCT. [file 40345_2022_269_MOESM1_ESM.tiff]
