# Supplementary material for: The effect of mindfulness-based cognitive therapy on rumination and a task-based measure of intrusive thoughts in patients with bipolar disorder
Source: Int J Bipolar Disord. 2022 Aug 12;10:22. doi: 10.1186/s40345-022-00269-1 (PMC9374865; doi:10.1186/s40345-022-00269-1)
Supplement: Supplementary file 2 — Additional file 2: Table S1. Sociodemographic and clinical characteristics, and baseline measures for patients that agreed or refused to perform the experimental tasks. Table S2. Sociodemographic and clinical characteristics, and baseline measures for patients who performed, or did not perform the experimental tasks. Table S3. Sociodemographic and clinical characteristics, and baseline measures for patients who completed baseline and post-treatment BFT versus patients who completed baseline BFT only. Table S4. No influence of time between measures of depressive/manic symptoms (T0), self-report questionnaires (T0a), and the BFT scores (T0b), on the association between those measures at baseline. Table S5. Bootstrapped BCa 95% confidence intervals of B for the regression model regarding the brooding subscale of the RRS: RRS_br.T1 – RRS_br.T0 = group + intercept. Table S6. Bootstrapped BCa 95% confidence intervals of B for the regression model regarding the number of negative intrusive thoughts: BFT.neg.T1 – BFT.neg.T0 = group + intercept. Table S7. Bootstrapped BCa 95% confidence intervals of B for the regression model regarding the number of positive intrusive thoughts: BFT.pos.T1 – BFT.pos.T0 = group + intercept. Table S8. Bootstrapped BCa 95% confidence intervals of B for the regression model regarding the number of neutral intrusive thoughts: BFT.neutral.T1 – BFT.neutral.T0 = group + intercept. Table S9. Bootstrapped BCa 95% confidence intervals of B for the regression model regarding the number of total intrusive thoughts: BFT.total.T1 – BFT.total.T0 = group + intercept. Table S10. Bootstrapped BCa 95% confidence intervals of B for the regression model regarding the emotion-focused subscale of the RPA: RPA_ER.T1 – RPA_ER.T0 = group + intercept. Table S11. Bootstrapped BCa 95% confidence intervals of B for the regression model regarding the self-focused subscale of the RPA: RPA_SR.T1 – RPA_SR.T0 = group + intercept [file 40345_2022_269_MOESM2_ESM.docx]

**Additional file 1: Figure S1. CONSORT Flow Diagram**

*Practical reasons consisted of limited number of research laptops; no research assistant available at every outpatients clinic, not enough time between baseline measurement and start MBCT.

**Additional file 2: Table S1**

Sociodemographic and clinical characteristics, and baseline measures for patients that agreed or refused to perform the experimental tasks

| Demographic characteristics | Subsample CT (*n*=52) | Refused task (*n*=22) | *Test statistic* | *p-value* |
| --- | --- | --- | --- | --- |
| Age, *Med (P25-P75)* | 46 (37 – 54)^1^ | 42 (37 – 51)^1^ | *U = 485.5,*  *z = - .620* | .535 **^c^** |
| Gender, *Female* (%) | 66.7^1^ | 71.4^1^ | *χ2*(1) = .155 | .786 **^a^** |
| Education | ^1^ | ^1^ | - | .855 **^b^** |
| - Low (%) | 11.8 | 4.8 |  |  |
| - Medium (%) | 29.4 | 33.3 |  |  |
| - High (%) | 57.7 | 61.9 |  |  |
| Married/living together (%) | 45.1^1^ | 47.6^1^ | *χ2*(1) = .038 | 1.000 **^a^** |
| Employed (%) | 41.2^1^ | 28.6^1^ | *χ2*(1) = 1.01 | .424 **^a^** |
| Clinical characteristics |  |  |  |  |
| Bipolar Type I (%) | 63.5 | 63.6 | *χ2*(1) = .00 | 1.000 **^a^** |
| Age first episode, *Med* (P25-P75) | 20.5 (17.5 – 25.0) | 20.0 (17.0 – 34.0) | *U = 533.5,*  *z = - .456* | .648 **^c^** |
| Number of episodes,  *Med* (P25-P75) | 15.5 (7.5 – 41.5) | 12.5 (7.0 – 24.0) | *U = 490.0, z = - .971* | .332 **^c^** |
| Outcome measures | ***M (SD)*** | ***M (SD)*** |  |  |
| Depressive symptoms (IDS-C) | 14.5 (12.6) | 15.7 (13.1) | *t*(72) = -.367 | .715 **^d^** |
| Manic symptoms (YMRS) | 1.8 (2.7) | 1.6 (1.7) | *t*(72) = .457 | .649 **^d^** |
| Rumination (RRS) | 11.3 (3.5)^4^ | 10.7 (3.1)^1^ | *t*(67) = .755 | .453 **^d^** |
| RPA_ER | 12.9 (2.8)^4^ | 12.4 (2.7)^1^ | *t*(67) = .765 | .447 **^d^** |
| RPA_SR | 8.6 (2.8)^4^ | 8.8 (3.1)^1^ | *t*(67) = -.146 | .880 **^d^** |
| *Note.* *IDS-C* = Inventory of Depressive Symptomatology - Clinician administered, *YMRS* = Young Mania Rating Scale, *RRS_br* = Brooding subscale of Ruminative Response Scale, *RPA_ER* = Emotion-Focused subscale of the Responses to Positive Affect questionnaire, *RPA_SR* = Self-Focused subscale of the Responses to Positive Affect questionnaire  ^a^ = χ^2^ test, ^b^ = Fisher’s Exact test, ^c^ Mann-Whitney test, ^d^  = Independent samples t test.  ^1,3^ Number of missing values: ^1^ = 1 missing, ^3^ = 3 missing | | | | |

**Additional file 2: Table S2**

Sociodemographic and clinical characteristics, and baseline measures for patients who performed, or did not perform the experimental tasks

| Demographic characteristics | RCT sample without tasks (*n*=92) | Subsample CT (*n*=52) | *Test-statistic* | *p-value* |
| --- | --- | --- | --- | --- |
| Age, Med (P25-P75) | 46 (37 – 59)^3^ | 46 (37 – 54)^1^ | *U = 2143*  *z = - .548* | .584 **^c^** |
| Gender, *Female* (%) | 59.6^3^ | 66.7^1^ | *χ2*(1) = .698 | .471 **^a^** |
| Education | ^3^ | ^1^ | *χ2*(1) = .449 | .784 **^a^** |
| - Low (%) | 9 | 11.8 |  |  |
| - Medium (%) | 33.7 | 29.4 |  |  |
| - High (%) | 55.4 | 57.7 |  |  |
| Married/living together (%) | 57.3^3^ | 45.1^1^ | *χ2*(1) = 1.94 | .218 **^a^** |
| Employed (%) | 30.3^3^ | 41.2^1^ | *χ2*(1) = 1.69 | .202 **^a^** |
| Clinical characteristics |  |  |  |  |
| Bipolar type I (%) | 51.1 | 63.5 | *χ2*(1) = 2.06 | .166 **^a^** |
| Age first episode, *Med* (P25-P75) | 20.0 (16.0 – 29.5)^1^ | 20.5 (17.5 – 25.0) | *U = 2306,*  *z = - .254* | .799 **^c^** |
| Number of episodes;  *Med* (P25-P75) | 14.0 (7.0 – 36.5) | 15.5 (7.5 – 41.5) | U = 2322,  z = - .291 | .771 **^c^** |
| Outcome measures | ***M* (SD)** | ***M* (SD)** |  |  |
| Depressive symptoms (IDS-C) | 16.0 (12.6) | 14.5 (12.6) | *t*(142) = .679 | .498 **^d^** |
| Manic symptoms (YMRS) | 2.1 (2.5) | 1.8 (2.7) | *t*(142) = .584 | .560 **^d^** |
| Rumination (RRS) | 11.5 (3.2)^3^ | 11.3 (3.5)^4^ | *t*(135) = .341 | .726 **^d^** |
| RPA_ER | 12.4 (2.9)^4^ | 12.9 (2.8)^4^ | *t*(134) = -1.00 | .316 **^d^** |
| RPA_SR | 8.7 (2.9)^4^ | 8.6 (2.8)^4^ | *t*(134) = .025 | .980 **^d^** |
| *Note. IDS-C* = Inventory of Depressive Symptomatology - Clinician administered, *YMRS* = Young Mania Rating Scale, *RRS_br* = Brooding subscale of Ruminative Response Scale, *RPA_ER* = Emotion-Focused subscale of the Responses to Positive Affect questionnaire, *RPA_SR* = Self-Focused subscale of the Responses to Positive Affect questionnaire  ^a^ = χ^2^ test, ^b^ = Fisher’s Exact test, ^c^ Mann-Whitney test, ^d^  = Independent samples t test.  ^1,3,4^ Number of missing values: ^1^ = 1 missing, ^3^ = 3 missing, ^4^ = 4 missing | | | | |

**Additional file 2: Table S3**

Sociodemographic and clinical characteristics, and baseline measures for patients who completed baseline and post-treatment BFT versus patients who completed baseline BFT only.

| Demographic characteristics | BFT completers (*n*=34) | BFT Baseline only (*n*=15) | *Test-statistic* | *p-value* |
| --- | --- | --- | --- | --- |
| Age, *Med (P25-P75)* | 49 (37 – 54) | 45.5 (38 – 53) | *U* = 197.5,  *z* = - .920 | .358 **^c^** |
| Gender, *Female* (%) | 61.8 | 85.7^1^ |  | .328 **^b^** |
| Education level |  |  |  | .588 **^b^** |
| - Low (%) | 14.7 | 7.1^1^ |  |  |
| - Medium (%) | 32.4 | 21.4^1^ |  |  |
| - High (%) | 52.9 | 71.4^1^ |  |  |
| Married/living together (%) | 58.8 | 42.9^1^ | *χ2*(1) = 1.02 | .313 **^a^** |
| Employed (%) | 41.2 | 57.1^1^ | *χ2*(1) = 1.95 | .163 **^a^** |
| Clinical characteristics |  |  |  |  |
| Bipolar type I (%) | 61.8 | 60.0 | *χ2*(1) = .014 | .91 **^a^** |
| Age first episode, *Med* (P25-P75) | 21.0 (18.0 – 25.0) | 18.0 (16.0 – 21.0) | *U* = 197.5,  *z* = - 1.25 | .210 **^c^** |
| Number of episodes;  *Med* (P25-P75) | 11.5 (7.0 – 44.0) | 24.0 (7.0 – 32.0) | *U* = 249.5,  z = - .119 | .905 **^c^** |
| Outcome measures | ***M* (SD)** | ***M* (SD)** |  |  |
| Depressive symptoms (IDS-C) | 15.9 (13.5) | 12.9 (11.3) | t(47) = - .738 | .464 **^d^** |
| Manic symptoms (YMRS) | 2.1 (3.0) | 1.6 (1.8) | t(47) = - .541 | .591 **^d^** |
| Rumination (RRS) | 11.5 (3.5)^2^ | 11.6 (3.6)^2^ | t(43) = .127 | .900 **^d^** |
| RPA_ER | 13.3 (2.7)^2^ | 12.5 (3.3)^2^ | t(43) = - .792 | .433 **^d^** |
| RPA_SR | 9.0 (2.6)^2^ | 8.4 (3.3)^2^ | t(43) = - .699 | .488 **^d^** |
| *Note. IDS-C* = Inventory of Depressive Symptomatology - Clinician administered, *YMRS* = Young Mania Rating Scale, *RRS_br* = Brooding subscale of Ruminative Response Scale, *RPA_ER* = Emotion-Focused subscale of the Responses to Positive Affect questionnaire, *RPA_SR* = Self-Focused subscale of the Responses to Positive Affect questionnaire  ^a^ = χ^2^ test, ^b^ = Fisher’s Exact test, ^c^ Mann-Whitney test, ^d^  = Independent samples t test.  ^1,2^ Number of missing values: ^1^ = 1 missing, ^2^ = 2 missing | | | | |

**Additional file 2: Table S4**

*No influence of time between measures of depressive/manic symptoms (T0), self-report questionnaires (T0a), and the BFT scores (T0b), on the association between those measures at baseline.*

|  | *df* | F | p-value |
| --- | --- | --- | --- |
|  | | | |
| *DV = IDS-C; IV’s = RRS_br, time between IDS-C (interview) and RRS_br (questionnaires)* | | | |
| Correct model | 3 | 3.837 | .016 |
| Intercept | 1 | .544 | .465 |
| RRS_br | 1 | 8.924 | .005 |
| Time IDS-C-RRS_br | 1 | .023 | .879 |
| RRS_br*Time IDS-C-RRS_br | 1 | .021 | .887 |
|  | | | |
| *DV = IDS-C; IV’s = negative intrusive thoughts (BFT), time between IDS-C (interview) and BFT* | | | |
| Correct model | 3 | 1.137 | .345 |
| Intercept | 1 | 7.975 | .007 |
| Negative intrusive thoughts | 1 | 3.123 | .084 |
| Time IDS-C-BFT | 1 | .713 | .403 |
| Negative intrusive thoughts*Time IDS-C-BFT | 1 | 1.907 | .174 |
|  | | | |
| *DV = RRS_br; IV’s = negative intrusive thoughts (BFT), time between RRS_br (questionnaires) and BFT* | | | |
| Correct model | 3 | 2.227 | .099 |
| Intercept | 1 | 135.7 | .000 |
| Negative intrusive thoughts | 1 | .749 | .392 |
| Time RRS_br-BFT | 1 | .154 | .697 |
| Negative intrusive thoughts*Time RRS_br-BFT | 1 | .059 | .810 |
|  | | | |
| *DV = positive intrusive thoughts (BFT); IV’s = YMRS, time between YMRS (interview) and BFT* | | | |
| Correct model | 3 | .892 | .453 |
| Intercept | 1 | 6.837 | .012 |
| YMRS | 1 | .163 | .689 |
| Time YMRS-BFT | 1 | .065 | .799 |
| YMRS*Time YMRS-BFT | 1 | .717 | .402 |
|  | | | |
| *DV = positive intrusive thoughts (BFT); IV’s = RPA_ER, time between RPA_ER (questionnaires) and BFT* | | | |
| Correct model | 3 | .513 | .675 |
| Intercept | 1 | .150 | .700 |
| RPA_ER | 1 | 1.347 | .253 |
| Time RPA_ER-BFT | 1 | .467 | .498 |
| RPA_ER*Time RPA_ER-BFT | 1 | .484 | .491 |
|  | | | |
| *DV = positive intrusive thoughts (BFT); IV’s = RPA_SR, time between RPA_SR (questionnaires) and BFT* | | | |
| Correct model | 3 | .176 | .912 |
| Intercept | 1 | .287 | .595 |
| RPA_ER | 1 | .447 | .508 |
| Time RPA_ER-BFT | 1 | .129 | .721 |
| RPA_ER*Time RPA_ER-BFT | 1 | .121 | .730 |
|  | | | |
| *DV = dependent variable; IV’s = independent variables; IDS-C = Inventory of Depressive Symptomatology - Clinician administered, YMRS = Young Mania Rating Scale, RRS_br = Brooding subscale of Ruminative Response Scale, RPA_ER = Emotion-Focused subscale of the Responses to Positive Affect questionnaire, RPA_SR = Self-Focused subscale of the Responses to Positive Affect questionnaire* | | | |

**Additional file 2: Table S5**

*Bootstrapped BCa 95% confidence intervals of B for the regression model regarding the brooding subscale of the RRS: RRS_br.T1 – RRS_br.T0 = group + intercept*

|  | *B* | Bias | SE | p-value | BCa 95% interval of *B* | |
| --- | --- | --- | --- | --- | --- | --- |
|  |  |  |  |  | **Lower** | **Upper** |
|  | | | | | | |
| *Bootstrap BCa linear regression 1* | | | | | | |
| Constant | 1.58 | -.005 | 1.32 | .25 | - 1.09 | 4.19 |
| Group (MBCT + TAU vs TAU) | - 2.11 | .005 | 0.92 | .035 | - 3.93 | - 0.33 |
| *Bootstrap BCa linear regression 2* | | | | | | |
| Constant | 1.58 | -.012 | 1.33 | .25 | - 1.25 | 4.32 |
| Group (MBCT + TAU vs TAU) | - 2.11 | .010 | 0.93 | .036 | - 3.92 | - 0.35 |
| *Bootstrap BCa linear regression 3* | | | | | | |
| Constant | 1.58 | .013 | 1.36 | .27 | - 1.13 | 4.23 |
| Group (MBCT + TAU vs TAU) | - 2.11 | -.008 | 0.94 | .038 | - 4.10 | - 0.28 |
| *Bootstrap BCa linear regression 4* | | | | | | |
| Constant | 1.58 | -.027 | 1.34 | .26 | - 1.08 | 4.01 |
| Group (MBCT + TAU vs TAU) | - 2.11 | .021 | 0.94 | .040 | - 4.13 | - 0.12 |
| *Bootstrap BCa linear regression 5* | | | | | | |
| Constant | 1.58 | -.010 | 1.35 | .26 | - 1.00 | 4.16 |
| Group (MBCT + TAU vs TAU) | - 2.11 | .010 | 0.94 | .038 | - 4.22 | - 0.22 |
|  | | | | | | |
| *Note. B* = unstandardized regression coefficient, *SE* = Standard Error, *BCa* = Bias-corrected and Accelerated  *BFT* = Breathing focus task, *T0* = Baseline, *T1* = Post-treatment, *MBCT* = Mindfulness-based Cognitive Therapy,  *TAU* = Treatment as usual, *RRS* = Ruminative Response Scale | | | | | | |

**Additional file 2: Table S6**

*Bootstrapped BCa 95% confidence intervals of B for the regression model regarding the number of negative intrusive thoughts: BFT.neg.T1 – BFT.neg.T0 = group + intercept.*

|  | *B* | Bias | SE | p-value | BCa 95% interval of *B* | |
| --- | --- | --- | --- | --- | --- | --- |
|  |  |  |  |  | **Lower** | **Upper** |
|  | | | | | | |
| *Bootstrap BCa linear regression 1* | | | | | | |
| Constant | 2.00 | .005 | 1.00 | .064 | 0.20 | 3.98 |
| Group (MBCT + TAU vs TAU) | - 1.47 | -.003 | 0.64 | .033 | - 2.76 | - 0.26 |
| *Bootstrap BCa linear regression 2* | | | | | | |
| Constant | 2.00 | .008 | 1.02 | .066 | 0.232 | 3.94 |
| Group (MBCT + TAU vs TAU) | - 1.47 | -.002 | 0.66 | .040 | - 2.90 | - 0.15 |
| *Bootstrap BCa linear regression 3* | | | | | | |
| Constant | 2.00 | -.010 | 1.02 | .068 | 0.17 | 3.93 |
| Group (MBCT + TAU vs TAU) | - 1.47 | .009 | 0.66 | .038 | - 2.85 | - 0.14 |
| *Bootstrap BCa linear regression 4* | | | | | | |
| Constant | 2.00 | -.033 | 0.99 | .061 | 0.27 | 3.70 |
| Group (MBCT + TAU vs TAU) | - 1.47 | .026 | 0.64 | .037 | - 2.87 | - 0.095 |
| *Bootstrap BCa linear regression 5* | | | | | | |
| Constant | 2.00 | .018 | 1.01 | .066 | 0.018 | 4.03 |
| Group (MBCT + TAU vs TAU) | - 1.47 | -.012 | 0.65 | .037 | - 2.70 | - 0.25 |
|  | | | | | | |
| *Note. B* = unstandardized regression coefficient, *SE* = Standard Error, *BCa* = Bias-corrected and Accelerated  *BFT* = Breathing focus task, *T0* = Baseline, *T1* = Post-treatment, *MBCT* = Mindfulness-based Cognitive Therapy,  *TAU* = Treatment as usual | | | | | | |

**Additional file 2: Table S7**

*Bootstrapped BCa 95% confidence intervals of B for the regression model regarding the number of positive intrusive thoughts: BFT.pos.T1 – BFT.pos.T0 = group + intercept*

|  | *B* | Bias | SE | p-value | BCa 95% interval of *B* | |
| --- | --- | --- | --- | --- | --- | --- |
|  |  |  |  |  | **Lower** | **Upper** |
|  | | | | | | |
| *Bootstrap BCa linear regression 1* | | | | | | |
| Constant | - 0.13 | -.005 | 1.32 | .92 | - 2.73 | 2.38 |
| Group (MBCT + TAU vs TAU) | 0.33 | .007 | 1.01 | .75 | - 1.61 | 2.55 |
| *Bootstrap BCa linear regression 2* | | | | | | |
| Constant | - 0.13 | .022 | 1.30 | .93 | - 2.80 | 2.54 |
| Group (MBCT + TAU vs TAU) | 0.33 | -.004 | 0.99 | .75 | - 1.45 | 2.29 |
| *Bootstrap BCa linear regression 3* | | | | | | |
| Constant | - 0.13 | .007 | 1.31 | .92 | - 2.65 | 2.41 |
| Group (MBCT + TAU vs TAU) | 0.33 | -.010 | 1.00 | .75 | - 1.52 | 2.29 |
| *Bootstrap BCa linear regression 4* | | | | | | |
| Constant | - 0.13 | -.013 | 1.33 | .93 | - 2.72 | 2.43 |
| Group (MBCT + TAU vs TAU) | 0.33 | .013 | 1.01 | .76 | - 1.48 | 2.33 |
| *Bootstrap BCa linear regression 5* | | | | | | |
| Constant | - 0.13 | .020 | 1.31 | .91 | - 2.69 | 2.45 |
| Group (MBCT + TAU vs TAU) | 0.33 | -.015 | 0.99 | .75 | - 1.52 | 2.33 |
|  | | | | | | |
| *Note. B* = unstandardized regression coefficient, *SE* = Standard Error, *BCa* = Bias-corrected and Accelerated  *BFT* = Breathing focus task, *T0* = Baseline, *T1* = Post-treatment, *MBCT* = Mindfulness-based Cognitive Therapy,  *TAU* = Treatment as usual | | | | | | |

**Additional file 2: Table S8**

*Bootstrapped BCa 95% confidence intervals of B for the regression model regarding the number of neutral intrusive thoughts: BFT.neutral.T1 – BFT.neutral.T0 = group + intercept*

|  | *B* | Bias | SE | p-value | BCa 95% interval of *B* | |
| --- | --- | --- | --- | --- | --- | --- |
|  |  |  |  |  | **Lower** | **Upper** |
|  | | | | | | |
| *Bootstrap BCa linear regression 1* | | | | | | |
| Constant | - 2.60 | -.035 | 1.2 | .055 | - 4.93 | - 0.47 |
| Group (MBCT + TAU vs TAU) | 1.93 | .023 | 0.84 | .047 | 0.29 | 3.78 |
| *Bootstrap BCa linear regression 2* | | | | | | |
| Constant | - 2.60 | .024 | 1.16 | .056 | - 5.10 | - 0.13 |
| Group (MBCT + TAU vs TAU) | 1.93 | -.023 | 0.84 | .048 | 0.47 | 3.45 |
| *Bootstrap BCa linear regression 3* | | | | | | |
| Constant | - 2.60 | -.004 | 1.19 | .054 | - 5.17 | - 0.30 |
| Group (MBCT + TAU vs TAU) | 1.93 | -.005 | 0.85 | .047 | 0.500 | 3.56 |
| *Bootstrap BCa linear regression 4* | | | | | | |
| Constant | - 2.60 | .002 | 1.16 | .052 | - 4.99 | - 0.333 |
| Group (MBCT + TAU vs TAU) | 1.93 | -.006 | 0.85 | .047 | 0.40 | 3.67 |
| *Bootstrap BCa linear regression 5* | | | | | | |
| Constant | - 2.60 | .009 | 1.18 | .051 | - 4.95 | - 0.31 |
| Group (MBCT + TAU vs TAU) | 1.93 | -.011 | 0.85 | .048 | 0.39 | 3.61 |
|  | | | | | | |
| *Note. B* = unstandardized regression coefficient, *SE* = Standard Error, *BCa* = Bias-corrected and Accelerated  *BFT* = Breathing focus task, *T0* = Baseline, *T1* = Post-treatment, *MBCT* = Mindfulness-based Cognitive Therapy,  *TAU* = Treatment as usual | | | | | | |

**Additional file 2: Table S9**

*Bootstrapped BCa 95% confidence intervals of B for the regression model regarding the number of total intrusive thoughts: BFT.total.T1 – BFT.total.T0 = group + intercept*

|  | *B* | Bias | SE | p-value | BCa 95% interval of *B* | |
| --- | --- | --- | --- | --- | --- | --- |
|  |  |  |  |  | **Lower** | **Upper** |
|  | | | | | | |
| *Bootstrap BCa linear regression 1* | | | | | | |
| Constant | - 0.73 | .023 | 1.60 | .67 | - 3.94 | 2.52 |
| Group (MBCT + TAU vs TAU) | 0.80 | -.009 | 1.17 | .51 | - 1.48 | 3.07 |
| *Bootstrap BCa linear regression 2* | | | | | | |
| Constant | - 0.73 | .016 | 1.60 | .66 | - 3.97 | 2.47 |
| Group (MBCT + TAU vs TAU) | 0.80 | -.010 | 1.18 | .53 | - 1.40 | 2.98 |
| *Bootstrap BCa linear regression 3* | | | | | | |
| Constant | - 0.73 | .017 | 1.58 | .66 | - 4.00 | 2.43 |
| Group (MBCT + TAU vs TAU) | 0.80 | -.017 | 1.15 | .51 | - 1.33 | 2.91 |
| *Bootstrap BCa linear regression 4* | | | | | | |
| Constant | - 0.73 | .005 | 1.60 | .67 | - 4.06 | 2.35 |
| Group (MBCT + TAU vs TAU) | 0.80 | -.007 | 1.17 | .52 | - 1.40 | 3.06 |
| *Bootstrap BCa linear regression 5* | | | | | | |
| Constant | - 0.73 | -.006 | 1.62 | .66 | - 3.89 | 2.39 |
| Group (MBCT + TAU vs TAU) | 0.80 | .002 | 1.19 | .53 | - 1.58 | 3.21 |
|  | | | | | | |
| *Note. B* = unstandardized regression coefficient, *SE* = Standard Error, *BCa* = Bias-corrected and Accelerated  *BFT* = Breathing focus task, *T0* = Baseline, *T1* = Post-treatment, *MBCT* = Mindfulness-based Cognitive Therapy,  *TAU* = Treatment as usual | | | | | | |

**Additional file 2: Table S10**

*Bootstrapped BCa 95% confidence intervals of B for the regression model regarding the emotion-focused subscale of the RPA: RPA_ER.T1 – RPA_ER.T0 = group + intercept*

|  | *B* | Bias | SE | p-value | BCa 95% interval of *B* | |
| --- | --- | --- | --- | --- | --- | --- |
|  |  |  |  |  | **Lower** | **Upper** |
|  | | | | | | |
| *Bootstrap BCa linear regression 1* | | | | | | |
| Constant | 0.71 | -.013 | 1.44 | .63 | - 1.80 | 3.31 |
| Group (MBCT + TAU vs TAU) | - 0.18 | .09 | 0.80 | .82 | - 1.99 | 1.56 |
| *Bootstrap BCa linear regression 2* | | | | | | |
| Constant | 0.71 | .008 | 1.45 | .63 | - 1.90 | 3.43 |
| Group (MBCT + TAU vs TAU) | - 0.18 | -.004 | 0.80 | .84 | - 1.85 | 1.47 |
| *Bootstrap BCa linear regression 3* | | | | | | |
| Constant | 0.71 | .015 | 1.44 | .63 | - 2.35 | 3.65 |
| Group (MBCT + TAU vs TAU) | - 0.18 | -.014 | 0.80 | .83 | - 1.61 | 1.22 |
| *Bootstrap BCa linear regression 4* | | | | | | |
| Constant | 0.71 | -.006 | 1.44 | .62 | - 2.06 | 3.53 |
| Group (MBCT + TAU vs TAU) | - 0.18 | .005 | 0.80 | .83 | - 1.81 | 1.45 |
| *Bootstrap BCa linear regression 5* | | | | | | |
| Constant | 0.71 | .013 | 1.47 | .63 | - 2.15 | 3.62 |
| Group (MBCT + TAU vs TAU) | - 0.18 | -.007 | 0.82 | .83 | - 1,79 | 1.42 |
|  | | | | | | |
| *Note. B* = unstandardized regression coefficient, *SE* = Standard Error, *BCa* = Bias-corrected and Accelerated  *BFT* = Breathing focus task, *T0* = Baseline, *T1* = Post-treatment, *MBCT* = Mindfulness-based Cognitive Therapy,  *TAU* = Treatment as usual, *RPA =* Responses to Positive Affect | | | | | | |

**Additional file 2: Table S11**

*Bootstrapped BCa 95% confidence intervals of B for the regression model regarding the self-focused subscale of the RPA: RPA_SR.T1 – RPA_SR.T0 = group + intercept*

|  | *B* | Bias | SE | p-value | BCa 95% interval of *B* | |
| --- | --- | --- | --- | --- | --- | --- |
|  |  |  |  |  | **Lower** | **Upper** |
|  | | | | | | |
| *Bootstrap BCa linear regression 1* | | | | | | |
| Constant | 2.39 | .015 | 1.45 | .11 | - 0.55 | 5.36 |
| Group (MBCT + TAU vs TAU) | - 1.59 | -.006 | 0.94 | .11 | - 3.40 | 0.23 |
| *Bootstrap BCa linear regression 2* | | | | | | |
| Constant | 2.39 | -.007 | 1.42 | .10 | - 0.17 | 5.01 |
| Group (MBCT + TAU vs TAU) | - 1.59 | .003 | 0.92 | .10 | - 3.50 | 0.37 |
| *Bootstrap BCa linear regression 3* | | | | | | |
| Constant | 2.39 | -.010 | 1.42 | .11 | - 0.49 | 5.24 |
| Group (MBCT + TAU vs TAU) | - 1.59 | .011 | 0.93 | .11 | - 3.35 | 0.25 |
| *Bootstrap BCa linear regression 4* | | | | | | |
| Constant | 2.39 | .012 | 1.43 | .12 | - 0.44 | 5.27 |
| Group (MBCT + TAU vs TAU) | - 1.59 | -.001 | 0.93 | .11 | - 3.32 | 0.22 |
| *Bootstrap BCa linear regression 5* | | | | | | |
| Constant | 2.39 | -.007 | 1.45 | .12 | - 0.74 | 5.11 |
| Group (MBCT + TAU vs TAU) | - 1.59 | .007 | 0.95 | .11 | - 3.50 | 0.41 |
|  | | | | | | |
| *Note. B* = unstandardized regression coefficient, *SE* = Standard Error, *BCa* = Bias-corrected and Accelerated  *BFT* = Breathing focus task, *T0* = Baseline, *T1* = Post-treatment, *MBCT* = Mindfulness-based Cognitive Therapy,  *TAU* = Treatment as usual, *RPA =* Responses to Positive Affect | | | | | | |
